# Application of APSIM model in winter wheat growth monitoring

**DOI:** 10.3389/fpls.2024.1500103

**Published:** 2024-11-14

**Authors:** Yunlong Tan, Enhui Cheng, Xuxiang Feng, Bin Zhao, Junjie Chen, Qiaoyun Xie, Hao Peng, Cunjun Li, Chuang Lu, Yong Li, Bing Zhang, Dailiang Peng

**Affiliations:** ^1^ School of Surveg and Land Information Engineering, Henan Polytechnic University, Jiaozuo, China; ^2^ Key Laboratory of Digital Earth Science, Aerospace Information Research Institute, Chinese Academy of Sciences, Beijing, China; ^3^ Aerospace Information Research Institute, Chinese Academy of Sciences, Beijing, China; ^4^ College of Resource and Environment, University of Chinese Academy of Sciences, Beijing, China; ^5^ International Research Center of Big Data for Sustainable Development Goals, Beijing, China; ^6^ School of Information Science and Engineering, Shandong Agricultural University, Taian, China; ^7^ School of Engineering, The University of Western Australia, Perth, WA, Australia; ^8^ Xinjiang Institute of Ecology and Geography, Chinese Academy of Sciences, Urumqi, Xinjiang, China; ^9^ Information Technology Research Center, Beijing Academy of Agriculture and Forestry Sciences, Beijing, China; ^10^ National Key Laboratory of Wheat Improvement and College of Agronomy, Shandong Agricultural University, Taian, China

**Keywords:** winter wheat, vegetation index, remote sensing, growth monitoring, cultivated land management

## Abstract

In the past, the use of remote sensing for winter wheat growth monitoring mainly relied on the relative growth assessment of a single vegetation index, such as normalized Vegetation index (NDVI). This study advanced the methodology by integrating field-measured data with Sentinel-2 data. In addition to NDVI, it innovatively incorporated two parameters, aboveground biomass (AGB) and leaf area index (LAI), for a more comprehensive relative growth assessment. Furthermore, the study employed the agricultural production systems simulator (APSIM) model to use LAI and AGB for absolute growth monitoring. The results showed that the simulated LAI and AGB closely match the field-measured values throughout the entire growth period of winter wheat under various conditions (R^2^ > 0.9). For relative growth monitoring, NDVI showed significant linear positive correlations (r > 0.74 and P< 0.05) with both LAI and AGB simulated by the APSIM model. Overall, this research shows that LAI and AGB obtained from the APSIM model provide a more detailed and accurate approach to monitoring of winter wheat growth. This improved monitoring capability can support effective land management arable and provide technical guidance to advance precision agriculture practices.

## 1 Introduction

In recent years, global climate change and dramatic shifts in land cover have posed significant challenges to the sustainable agricultural development. The increasing risk of food shortages has intensified global concerns about food security (Godfray et al., 2010; Atzberger, 2013; Battude et al., 2016). Wheat, one of the three staple grains, is cultivated worldwide. Its production, marketing, processing and consumption are closely integrated into daily life (Jiang et al., 2021). Providing over 20% of the energy needs for nearly half of the world’s population, wheat is a critical food source (Shiferaw et al., 2013). It is cultivated on approximately 30.7% of the global cereal acreage, making it the most widely grown cereals, surpassing corn, rice, and soybeans in cultivation area (Zhao et al., 2018). Timely and accurate assessment of winter wheat growth conditions and yield prediction are essential for shaping agricultural policies, informing market strategies, and ensuring national food security (Jin et al., 2018). As a result, crop growth monitoring is a significant research area, with remote sensing and crop growth simulation being particularly promising fields experiencing for practical application.

Crop growth monitoring encompasses both relative and absolute assessments, providing a scientific basis for monitoring crop growth conditions and forecasting yield by examining various growth parameters and their interrelationships (Wu et al., 2004; Xie et al., 2019). Relative growth monitoring involves comparing the current year’s growth with the same period from the previous year, offering insights into how winter wheat growth compares over time (Wu and Yan, 2002; Wu et al., 2004; Shi et al., 2015; Rao et al., 2021). For example, Yin et al. employed multi-source data to compare key growth stages across two years (Yin et al., 2021), highlighting growth trends. Sun et al. leveraged the NDVI difference model to analyze winter wheat growth patterns (Sun et al., 2020). While remote sensing technology allows for broad, macro-level monitoring of crop growth across years, it often lacks the detail needed for within-crop changes (Huang et al., 2019; Lan et al., 2019; Zhang et al., 2021). In contrast, absolute growth monitoring provides a direct assessment of winter wheat’s growth status and yield potential by measuring key growth indicators, such as LAI and AGB (A and Xu, 2021; Chang et al., 2023; Wang et al., 2023d). This method is well-suited for in-depth investigations of specific areas or crop types. Although absolute growth monitoring can be highly precise under experimental conditions, it requires substantial labor and financial resources for extensive field data collection, rendering it less feasible for large-scale monitoring efforts (Wang et al., 2022; Ma et al., 2023). Most studies focus on relative growth monitoring (Wu et al., 2017; Wang et al., 2023a; Zhu et al., 2023b), with fewer integrating both relative and absolute growth methods to monitor winter wheat growth.

Crop growth monitoring methods can be primarily categorized into two types: remote sensing data-based methods and growth model simulation-based methods. Remote sensing offers the ability to periodically acquire extensive surface crop data, providing quantitative assessments of crop growth on a regional scale through appropriate inversion methods (Wang et al., 2023b; Hong et al., 2024; Wang et al., 2024). In recent years, remote sensing has been increasingly used for crop classification, nutrient diagnosis, growth assessment, and disease monitoring, offering valuable insights for agricultural management and market decision-making. Compared to traditional information-gathering methods, remote sensing offers distinct advantages in monitoring and characterizing the physiological and biochemical parameters of crops (Chen et al., 2016). Since the 1970s, remote sensing techniques have been employed to monitor crop growth, and today, the technology and methodologies for wheat growth monitoring using satellite remote sensing data have become increasingly advanced. Most wheat growth monitoring via remote sensing primarily relies on the relationship between vegetation indices and agricultural parameters to develop regression models. However, this approach heavily depends on vegetation indices, with limited uses of other growth monitoring parameters, such as LAI and AGB (Luo et al., 2005; Yu et al., 2012; Su et al., 2019; Lu et al., 2020). In contrast, crop growth models use mathematical models to describe the growth and development of crops based on weather, soil, crop variety characteristics, and crop management practices. These models are grounded in principles of material balance and energy conservation, leveraging computer technology to systematically simulate key physiological processes such as photosynthesis, respiration, and transpiration. By establishing mathematical models to simulate crop growth at fixed time intervals, these models offer a robust framework for space-time analysis and continuity. Crop growth modeling can simulate the dynamics of crop growth at a specific point scale and provide mechanistic explanations for variations in crop growth and yield. Currently, over 200 crop models are available globally, with some of the most commonly used being the DSSAT, APSIM, and WOFOST models. The DSSAT model is capable of simulating the growth cycle, maturity process and yield formation of various crops (Yang et al., 2012; Liu et al., 2013; Wang et al., 2023d). The WOFOST (Zhuo et al., 2022a, b, 2023) model is recognized for its focus on soil and climate conditions and its ability to operate under multiple constraints. The APSIM (Briak and Kebede, 2021; He, 2022; Winn et al., 2023) model is extensively utilized and validated, taking into account factors such as soil quality, crop ecological processes, and atmospheric conditions (Wang et al., 2007). Its comprehensive approach makes it particularly suitable for assessing crop growth under various management practices.

Given the limitations of relying solely on relative growth monitoring—such as inadequate capture of detailed crop changes—as well as the current reliance on vegetation indices in most winter wheat monitoring studies, this research integrates the APSIM crop model with Sentinel-2 data. By incorporating AGB and LAI alongside NDVI, this study aims to provide a more comprehensive analysis through relative and absolute growth monitoring. The goal is to enable more detailed and accurate monitoring of winter wheat growth, ensure timely detection of abnormal conditions, optimize management and decision-making processes, ultimately enhancing both ecological and economic outcomes.

## 2 Materials and methods

### 2.1 Study areas

**Figure 1:**
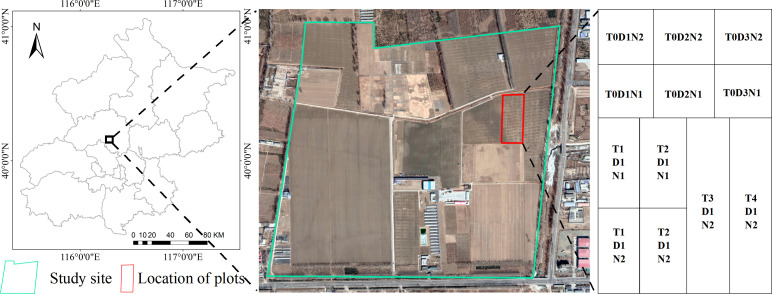
Overview of the study area and field trial design in Beijing.

As shown in **Figure 1**, field experiments in our study were conducted from 2021 to 2023 at the Precision Agriculture Demonstration Base of the National Research Center for Agricultural Information Technology, located in Xiaotangshan Township, Changping District, Beijing, China. The region experiences an average annual temperature ranging between 10 and 13 degrees Celsius, with average daily sunshine hours spanning from 6 to 9 hours. Precipitation in the area is distributed unevenly throughout the seasons. The experimental crop was winter wheat, with a growth cycle commencing in late October and concluding with the harvest in mid-June of the following year. The soil type belongs to the widely distributed tidal soil in North China.

### 2.2 Field experiment design

The experiment was conducted out from 2021 to 2023 at the experimental base in Xiaotangshan, Beijing, with an experimental field area of 240 m×75 m. The experiment was set up in 12 plots, and the specific plot distribution is shown in **Figure 1**. The test crop in the 2021-2022 test field is winter wheat (Jingdong 18), with a sowing depth of 3 cm and a sowing row spacing of 16.7 cm. The sowing time is October 11, 2021, and the harvest time is June 18, 2022. The seeding rate was set to three treatments: normal sowing D1 (375kg/ha), 50% seeding rate D2 (187.5kg/ha) and 75% seeding rate D3 (281.25kg/ha). Two treatments of normal fertilization (N2) and normal fertilization halved (N1) were used for fertilization. The normal fertilization (N2) was based on the application of the base fertilizer phosphate fertilizer diamine 450 kg/ha (containing N and P_2_O_5_ accounted for 18% and 46%, respectively) at the time of sowing, and the urea 240 kg/ha (containing N accounted for 46%) was applied when the winter wheat returned to green in the next year as a topdressing. The normal fertilization amount was halved (N1) according to the application of base fertilizer 225kg/ha phosphate diamine (including N and P_2_O_5_ accounted for 18% and 46%, respectively) at sowing, and urea 120 kg/ha (including N accounted for 46%) was applied as topdressing when winter wheat turned green in the next year. Five tillage methods were adopted: no tillage (T0), subsoiling tillage (T1), plow tillage (T2), rotation (T3) (plow tillage from 2021 to 2022, rotary tillage from 2022 to 2023) and rotary tillage (T4). Other field management conditions were the same in each plot. The measured data acquisition time for 2021-2022 is shown in **Table 1**.

**Table 1:** The collection time of measured data and image data from 2021 to 2023.

Data type	Acquisition time	Data type	Acquisition time	Time interval between measured data and Sentinel-2 image data(day)
	2022/4/08		2022/4/04	4
	2022/4/29		2022/5/02	3
Field measured data	2022/5/20	Sentinel-2	2022/5/22	2
	2023/3/21		2023/3/18	3
	2023/4/08		2023/4/07	1
	2023/5/08		2023/5/07	1

The test crop was winter wheat (Jingdong 18) in the test field from 2022 to 2023, with a sowing depth of 3 cm and a sowing row spacing of 16.7 cm. The sowing time is October 10,2021, and the harvest time is June 15,2022. The sowing amount and fertilization design are the same as those in 2021-2022. In addition to rotation, the same tillage methods as in 2021-2022 are adopted: no-tillage (T0), subsoiling (T1), plow tillage (T2), rotation (T3) (plow tillage in 2021-2022, rotary tillage in 2022-2023) and rotary tillage (T4). The other field management conditions are the same in each plot. The measured data collection time in 2022-2023 is shown in **Table 1**.

### 2.3 Data acquisition

#### 2.3.1 Field measured data

The precision of soil parameters directly influences the predictive accuracy of crop growth models, as these parameters are crucial determinants of crop growth and development. In this study, five soil profile parameters were measured across depths of 0 to 20 cm, 20 to 40 cm, 40 to 60 cm, 60 to 80 cm, and 80 to 100 cm, with initial soil water and nutrient values presented in **Table 2**. Soil parameters and other relevant data for the study area were obtained from the Xiaotangshan Experimental Base and related literature, included measurements of saturated water content, wilting coefficient, and field water holding capacity at various depths. The saturated water content was determined by collecting soil samples from different layers, placing them in containers, and gradually adding water until the soil reached a fully saturated state—indicated by the formation of a layer of free-flowing water on the soil surface. At this point, the soil moisture content was recorded. The wilting coefficient was obtained by place the soil samples in a greenhouse or controlled climate chamber, allowing the soil to dry fully to the permanent wilting point of the plant. The moisture content at this stage represents the wilting coefficient for that specific depth. Field capacity was determined by generating the soil moisture characteristic curve using a measuring instrument and reading the water content corresponding to the field water holding state from the curve. Specific soil parameters are shown in **Table 2**.

**Table 2:** Soil parameters.

Depth(cm)	Volume weight of soil(g·cm^-3^)	Saturation capacity(cm^3^·cm^-3^)	Wilting coefficient(cm^3^·cm^-3^)	Field capacity(cm^3^·cm^-3^)
0-20	1.32	0.48	0.10	0.34
20-40	1.43	0.46	0.12	0.33
40-60	1.43	0.46	0.13	0.32
60-80	2.04	0.47	0.15	0.31
80-100	1.93	0.47	0.14	0.31

In this study, two methods were employed to calculate and collect LAI: the specific leaf weight method and LAI-2200. The specific leaf weight method is particularly suitable for basic scenarios and standard measurement conditions, while the LAI-2200 is ideal for research and applications requiring higher accuracy and faster measurements. Both methods have their own advantages in different application scenarios. LAI data were obtained by measuring at five different locations within a 100 m^2^ plot using the LAI-2200 device, with the average of these measurements calculated to represent the LAI value for the plot. The leaf weight method collects the winter wheat in an area of 0.5m×0.5m and takes the sample of a certain area to randomly select a number of leaf blades and then takes the leaf blades in the width of the narrower more consistent place to cut the length of the small section of 2 or 3 cm. After that, get the width of the blade. Then the area can be calculated. The leaves are dried and weighed. The specific calculations are performed according to the following formula:


(1) $$LAI=\frac{W_{1}+W_{2}}{A*W_{1}}*S*m$$

where *A* is the total sample area, m is the number of plants or tillers, *S* is the selected small part of the sample area, *W*_1_ is the quality of the selected small part of the sample after drying, and *W*_2_ is the quality of the remaining green leaves after drying.

The collection step of aboveground biomass is to select 20 winter wheat plants with uniform growth in the measurement range, separate different plants according to organs (stems, leaves, ears), kill them at 105**°**C for 30** **min, and dry them at 85**°**C to constant weight. The organs are weighed separately, and the sum is the dry matter weight of the plant, which is recorded as the dry weight of the aboveground part of the plant, and the dry biomass per unit land area is calculated according to the density.

#### 2.3.2 Meteorological data

**Figure 2:**
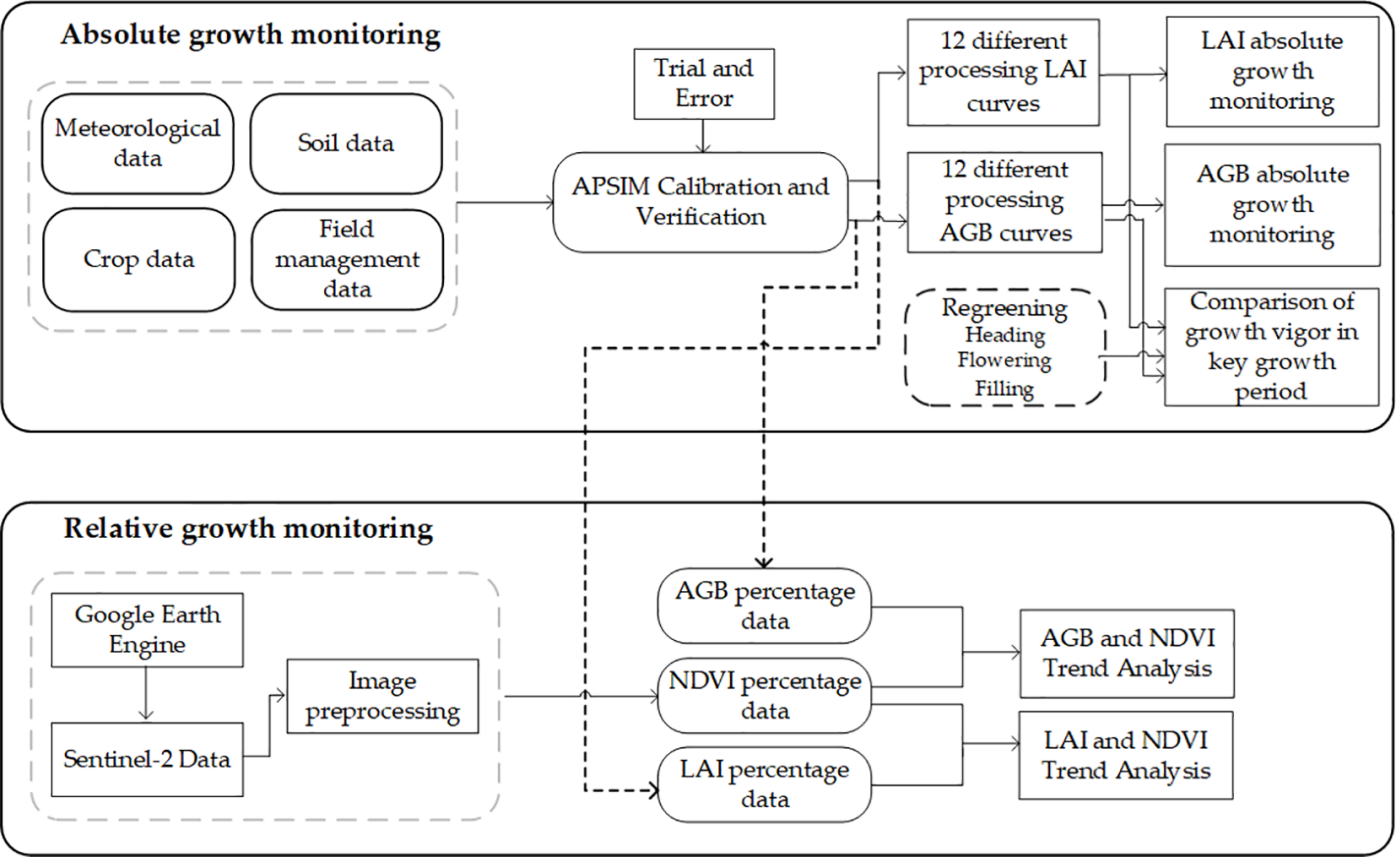
Technical route.

As shown in **Figure 2**, APSIM models rely on meteorological data as the basic input for their operation, which include daily average temperature and daily radiation. The climate data of the Xiaotangshan area are derived from the meteorological products provided by the European Centre for Medium-Range Weather Forecasts (https://cds.climate.copernicus.eu/). The spatial resolution is 0.25°×0.25°, and the time resolution is hourly. The daily precipitation (mm), daily radiation (MJ/m^2^), daily maximum temperature (°C), daily minimum temperature (°C), and daily potential evaporation (mm) are input into the weather model.

#### 2.3.3 Remote sensing data

This study uses Sentinel-2 data from the GEE cloud platform. The Google Earth Engine (GEE) platform offers Sentinel-2 satellite images in two product types, determined by the level of preprocessing applied. Compared with TOA data, SR data have better data consistency and stability in time series. Given the revisit cycle, images were chosen to align as closely as possible with the timing of the field-measured data collection. The acquisition times for both the field measured data and the image data are presented in **Table 1**.

### 2.4 APSIM model and calibration

APSIM is a mechanism model that uses a general growth process to simulate the development and growth of crops (Huang et al., 2018; Zhu et al., 2023a). The model integrates various sub-modules—including crop, soil, management, and meteorology—into a modular framework, with meteorological data, crop data, soil data, and field management practices input into their respective sub-modules. Meteorological data, including precipitation, radiation, maximum and minimum temperatures, and potential evaporation, are used to drive the model, and assess crop responses and adaptations to climate change. Crop growth data encompass the phenological development of crops, such as growth stage scales, accumulated temperature calculations, and photosynthesis algorithms, and help the model simulate the entire crop life cycle from sowing to harvest. Soil data, which include the physical and chemical properties of the soil, are crucial for simulating root growth as well as water and nutrient uptake. Field management data, such as sowing time, sowing density, and fertilizer application, this allows the model to analyze the potential impacts of various management strategies on crop growth and yield.

The model must go through a series of debugging and calibration before application (Liu et al., 2023). Therefore, this study selected the data from 2021-2022 to calibrate the model, and verified the model through the data from 2022-2023. In the model calibration, this study went through the following steps. First, basic data such as meteorology, soil, and field management were input to run the model. Secondly, the LAI and AGB and other simulation results of the model operation were compared with the measured data, and the trial and error method, which is currently more common in model calibration, was used to gradually adjust the sensitive parameters until the measured data of different test schemes matched the simulation results (Liang et al., 2016; Chen et al., 2019; Jin et al., 2021; Wang et al., 2023c). Finally, the parameter values were determined to complete the model calibration. Regarding the selection of sensitive parameters, because the sensitive parameters of models in different climate regions are different, this study selected literature with the same or similar distance to the experimental area, and selected parameters that have a greater impact on the model results from the literature. Based on this standard, sensitive parameters were selected, and finally 5 crop parameters that are more sensitive to leaf area index and aboveground biomass were selected (Liu et al., 2011; He and Zhao, 2015; Xing et al., 2017b; Zhang et al., 2023). As shown in **Table 3**.

**Table 3:** Parameters after calibration.

Crop	Parameter	Lower bound	Upper bound	Initial Settings	Parameters after calibration
	vern_sens	0	5	1.5	3.1
	photop_sens	0	5	3.0	3.5
Wheat	Startgf_to_mat(°C/d)	200	900	580	550
	Potential_grain_filling_rate(mg/d)	0.001	0.005	0.0027	0.0029
	grains_per_gram_stem(grain/d)	10	40	27.6	29.5
	max_grain_size(g)	0.02	0.06	0.048	0.046

### 2.5 Method

#### 2.5.1 Absolute growth monitoring of winter wheat based on APSIM model

The accuracy of LAI and AGB of winter wheat simulated by the APSIM model was assessed through comparing the simulated values against the measured values. The determination coefficient (R^2^), root mean square error (RMSE) and normalized root mean square error (NRMSE) were used as the evaluation criteria for the growth results, calculated using Equations 2-4. The absolute growth range of LAI and AGB was compared across four key growth periods. In this paper, the specific growth periods of winter wheat, as determined by both domestic and international literature and experience, are presented in **Table 4**. The key growth stages selected for the relative growth study include regreening, heading, flowering, and filling. The technical route of this study is shown in **Figure 2**.

**Table 4:** Key growth period of winter wheat.

Item	Phenological phase									
	Emergence	Tillering	Wintering	Regreening	Jointing	Booting	Heading	Flowering	Filling	Maturity
2022/2023	10/16	11/2	1/4	3/18	4/13	4/25	5/5	5/15	5/26	6/10


(2) $$R^{2}=\frac{\sum_{i=1}^{\text{n}}{\left(X_{i}-\overset{-}{X}\right)}^{2}{\left(Y_{i}-\overset{-}{Y}\right)}^{2}}{n\sum_{i=1}^{n}{\left(X_{i}-\overset{-}{X}\right)}^{2}\sum_{i=1}^{n}{\left(Y_{i}-\overset{-}{Y}\right)}^{2}}$$

(3) $$RMSE=\sqrt{\frac{\sum_{i=1}^{n}{\left({\text{Y}}_{i}-X_{i}\right)}^{2}}{n}}$$

(4) $$NRMSE=\frac{RMSE}{\overset{-}{X}}\times 100\%$$

where *n* denotes the number of samples, *X*_i_, $\overset{-}{X}$ , *Y*_i_, $\overset{-}{Y}$ represent the actual observation data, the actual observation data mean, the model’s prediction data, and the model prediction data mean, respectively.

#### 2.5.2 Relative growth monitoring of winter wheat based on APSIM model combined with remote sensing data

To study the relative growth of crops, vegetation indices provide crucial information for monitoring crop growth dynamics. Common vegetation indices include RVI (Gonenc et al., 2019), NDVI (Huang et al., 2021), DVI (Gunathilaka, 2021), and EVI (Shammi and Meng, 2021), etc. Of these, NDVI is the most widely used index for assessing vegetation conditions, as it is particularly sensitive to the growth status of vegetation and can directly reflect its health. The NDVI calculation formula is as follows:


(5) $$NDVI=\frac{NIR-R}{NIR+R}$$

where *NIR* corresponds to the B8 band of the Sentinel-2 data, and *R* corresponds to the B4 band of the Sentinel-2 data.

In this paper, Sentinel-2 data is used to monitor the relative growth of winter wheat by using NDVI and APSIM models. We analyzed the correlation between NDVI, LAI, and AGB using Pearson’s correlation coefficient. NDVI data for six periods from 2021 to 2023 were obtained from the study area. For each of the three periods in 2022, the corresponding NDVI data from 2023 were subtracted and then divided by the 2022 NDVI data to calculate nine sets of NDVI percentage differences. In addition, the same method was used to calculate the difference percentage data between LAI and AGB output by APSIM model respectively The calculation method is shown in Formula 6. Pearson correlation analysis was then performed using the NDVI percentage difference data along with the percentage difference data for LAI and AGB. Through the Pearson correlation coefficient and P-value, the trends in relative growth in the study area were analyzed. Pearson’s correlation coefficient was calculated as shown in Equation 7.


(6) $$X_{\text{percentage data}}=1-\frac{2023X_{\text{b}}}{2022X_{\text{a}}}$$

Where *X* represents NDVI, LAI, or AGB, a represents the three measured data dates in 2022, and b represents the three measured data dates in 2023.


(7) $$\text{r}=\frac{\sum_{i=1}^{n}\left(X_{i}-\overset{-}{X})(Y_{i}-\overset{-}{Y}\right)}{\sqrt{\sum_{i=1}^{n}{\left(X_{i}-\overset{-}{X}\right)}^{2}}\sqrt{\sum_{i=1}^{n}{\left(Y_{i}-\overset{-}{Y}\right)}^{2}}}$$

where *X*_i_ represents the value of remote sensing NDVI of the ith sample, $\overset{-}{X}$ is the average value of remote sensing NDVI sample, *Y*_i_ is the model simulation value of LAI or AGB of sample *i*, $\overset{-}{Y}$ is the average value of LAI or AGB model simulation, n is the number of samples.

After calculating the correlation coefficient *r*, it needs to be tested whether it is statistically significant, i.e., whether it is significant or not, and its formula is shown in (8). Consult the t distribution to determine the corresponding P value.


(8) $$\text{t}=\frac{r\sqrt{n-2}}{\sqrt{1-r^{2}}}$$

## 3 Results

### 3.1 Absolute growth monitoring of winter wheat based on LAI and AGB model simulation data

#### 3.1.1 Absolute growth monitoring of winter wheat based on LAI model simulation data

**Figure 3:**
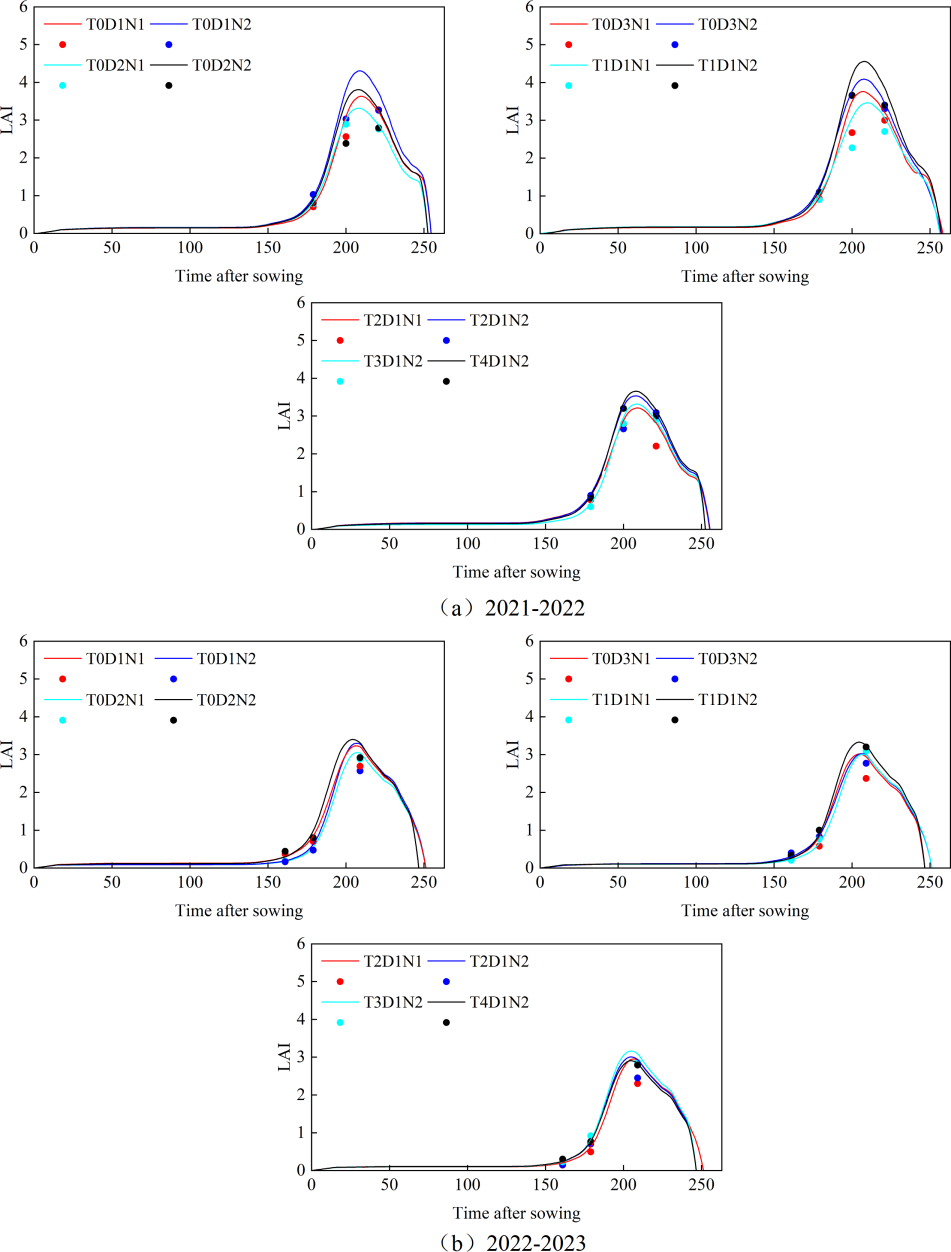
**(A)** Absolute growth monitoring of winter wheat LAI simulated by APSIM model under different tillage methods, fertilization and sowing treatments from 2021 to 2022; **(B)** Absolute growth monitoring of winter wheat LAI simulated by APSIM model under different tillage methods, fertilization and sowing treatments from 2022 to 2023. The curve represents the simulated value, and the point represents the measured value.

**Figure 3** shows the dynamic change changes in LAI as the growth index under varying fertilization, sowing, and tillage treatments during in 2021-2022 and 2022-2023 growing seasons. In this study, experimental data from the 2021 to 2022 winter wheat season were used to calibrate the model parameters, while data from the 2022 to 2023 season were employed for model validation. The analysis focused on the LAI growth of winter wheat in Xiaotangshan from 2021 to 2023. As the winter wheat growth period progressed, the LAI remained relatively stable during the overwintering phase. After regreening (about 170 d), winter wheat entered a phase of rapid growth, with LAI peaking towards the end of the growth period (210 d). Following the filling stage, LAI began to decline, approaching zero by the end of the filling period (about 250 d). Although the LAI values in different periods are different under different fertilization rates, seeding rates, and tillage methods, they all have a common growth trend, from regreening to maturity, LAI showed a parabolic trend. From the results of absolute growth monitoring in 2021-2023: (1) Other conditions are consistent, under different fertilization conditions. N2 (normal fertilizer rate) had relatively higher LAI and better growth, and halving the fertilizer rate resulted in relatively smaller leaf area of winter wheat plants, with a reduction of 0.12 to 1.08 in the peak LAI of the model simulation. (2) Other conditions are the same, under different seeding rates. From 2021 to 2022, D1 (normal seeding rate) grew better than D2 (50% seeding rate), and LAI was higher. However, when the fertilizer was halved, from 2021 to 2022, D3 (75% seeding rate) grew better than D1 (normal seeding rate) and D2 (50% seeding rate), and from 2022 to 2023, D3 (75% seeding rate) and D1 (normal seeding rate) grew similarly. The reason may be that the appropriate reduction of seeding rate leads to the improvement of light and nutrient utilization efficiency and thus promotes growth. (3) Other conditions are the same, under different tillage methods, the LAI of winter wheat in 2021-2022 shows T1 > T4 > T2 > T3, and the LAI of winter wheat in 2022-2023 shows T1 > T3 > T4 > T2. Subsoiling tillage showed better growth results than other tillage methods. From 2021 to 2022, the determination coefficient R^2^ for growth results using LAI as the growth index under different tillage, fertilization, and sowing treatments ranged from 0.85 to 0.98, with RMSE values between 0.02 and 0.51, and NRMSE values ranging from 1.1% to 18.3%. From 2022 to 2023, the R^2^ values ranged from 0.92 to 0.98, RMSE from 0.03 to 0.22, and NRMSE from 1.4% to 15%. The meaning of the field number is shown in **Table 5**.

**Table 5:** The specific meaning of field number.

The plot number	Processing mode	The plot number	Processing mode
T0D1N2	No-tillage, normal sowing rate, normal fertilization rate	T1D1N1	Subsoiling tillage, normal sowing rate, half of the amount of fertilizer
T0D2N2	No-tillage, 50% seeding rate, normal fertilizer rate	T1D1N2	Subsoiling tillage, normal sowing rate, normal fertilization rate
T0D3N2	No-tillage, 75% seeding rate, normal fertilizer rate	T2D1N1	Plow tillage, normal seeding rate, half the amount of fertilizer
T0D1N1	No-tillage, normal sowing rate, half of the amount of fertilizer	T2D1N2	Plow tillage, normal sowing amount, normal fertilization amount
T0D2N1	No-tillage, 50% seeding rate, half of the amount of fertilizer	T3D1N2	Rotation tillage, normal sowing rate, normal fertilization rate
T0D3N1	No-tillage, 75% seeding rate, half of the amount of fertilizer	T4D1N2	Rotary tillage, normal sowing rate, normal fertilization rate

**Figure 4:**
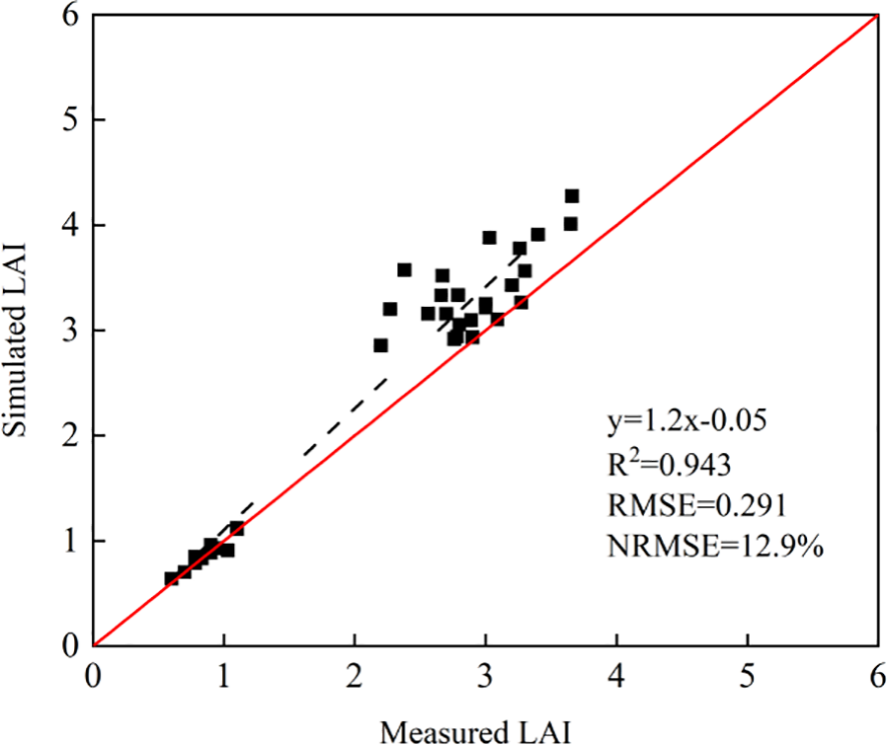
Evaluation of LAI growth accuracy from 2021 to 2023.

As shown in **Figure 4**, the accuracy results of absolute growth monitoring with LAI as the growth index were given under different fertilization rates, seeding rates, and tillage methods in 2021-2023. The R^2^ of simulated LAI in 2021-2023 was 0.943, and the simulated LAI was in good agreement with the measured LAI. The RMSE was 0.291, and the NRMSE was 12.9%.

#### 3.1.2 Absolute growth monitoring of winter wheat based on AGB model simulation data

**Figure 5:**
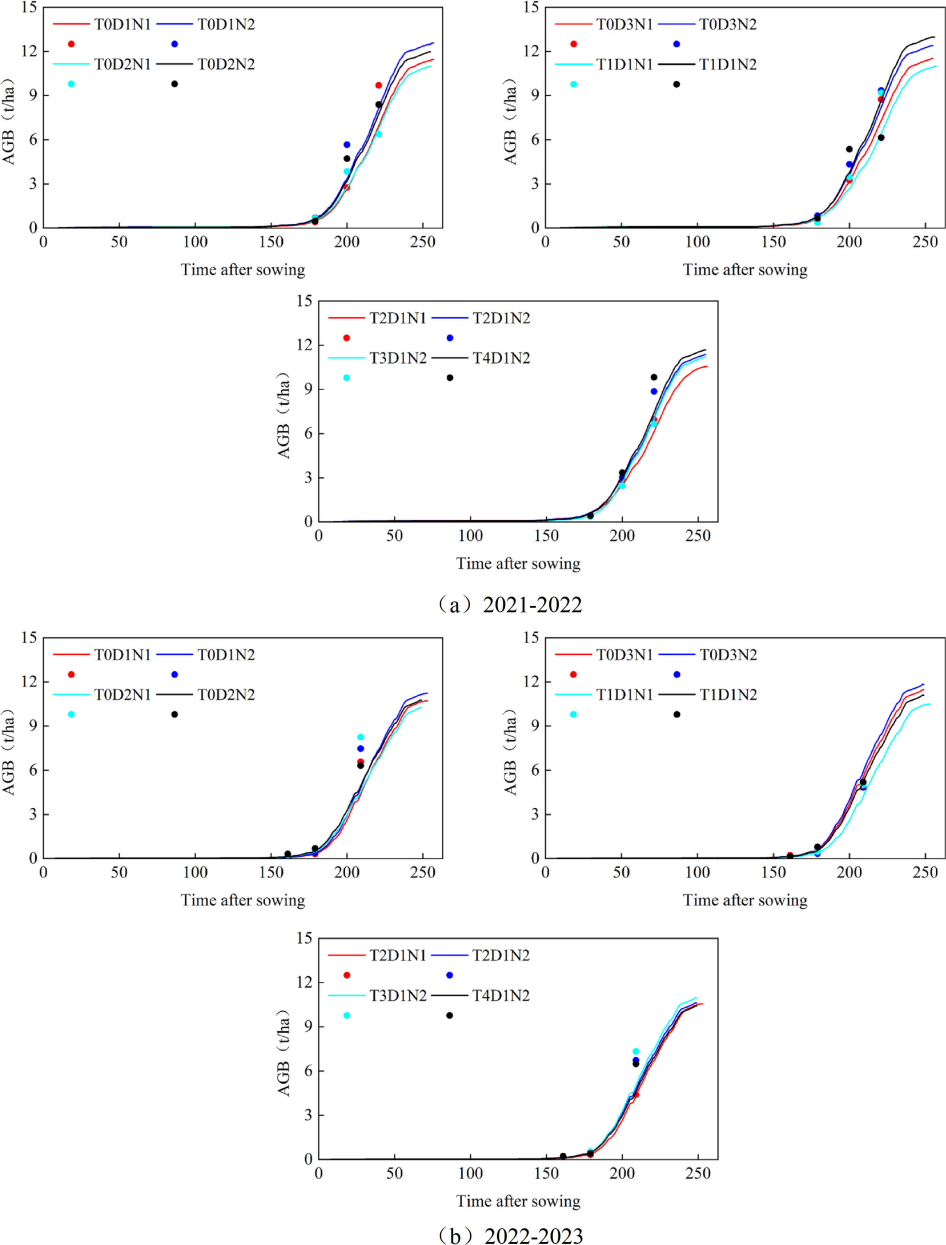
**(A)** Absolute growth monitoring of winter wheat AGB simulated by APSIM model under different tillage methods, fertilization and sowing treatments from 2021 to 2022; **(B)** Absolute growth monitoring of winter wheat AGB simulated by APSIM model under different tillage methods, fertilization and sowing treatments from 2022 to 2023. The curve represents the simulated value, and the point represents the measured value.

As shown in **Figure 5**, the dynamic change results of AGB under different fertilization, seeding amount and tillage methods in 2021-2022 and 2022-2023. Throughout the winter wheat growth period, the APSIM model simulated a general upward trend in AGB, with the growth rate significantly accelerating after the regreening period (about 175 d). The growth rate peaked near the end of the growth period (about 210d), after which it gradually slowed, with biomass accumulation ceasing by the end of the filling stage (about 250 d). 2021-2023, from the results of absolute growth monitoring: (1) Other conditions are consistent, under different fertilization conditions. From 2021 to 2022, the AGB of N2 (normal fertilization) was relatively high and the growth was better. The reason may be that the halving of fertilization amount makes the imbalance of nutrient supply and demand of winter wheat plants and the chlorophyll content of leaves decrease, and the photosynthesis ability decreases, thereby reducing the accumulation of AGB. (2) Other conditions are the same, under different seeding rates. From 2021 to 2022, D1 (normal seeding rate) grew better than D2 (50% seeding rate), but from 2021 to 2023, D3 (75% seeding rate) grew better than D1 (normal seeding rate). (3) Other conditions are the same, under different tillage methods. The AGB of winter wheat in 2021-2022 showed T1 > T4 > T2 > T3, and the AGB of winter wheat in 2022-2023 showed T1 > T3 > T2 > T4. Subsoiling tillage showed better growth results than other tillage methods. From 2021 to 2022, the determination coefficient R^2^ for growth results using AGB as the growth index under different tillage, fertilization, and sowing treatments ranged from 0.85 to 0.95, with RMSE values between 0.18 t/ha to 1.75 t/ha, and NRMSE values ranging from 1% to 35.6%. From 2022 to 2023, the R^2^ values ranged from 0.9 to 0.97, RMSE from 0.15 t/ha to 0.75 t/ha, and NRMSE from 3% to 9%.

**Figure 6:**
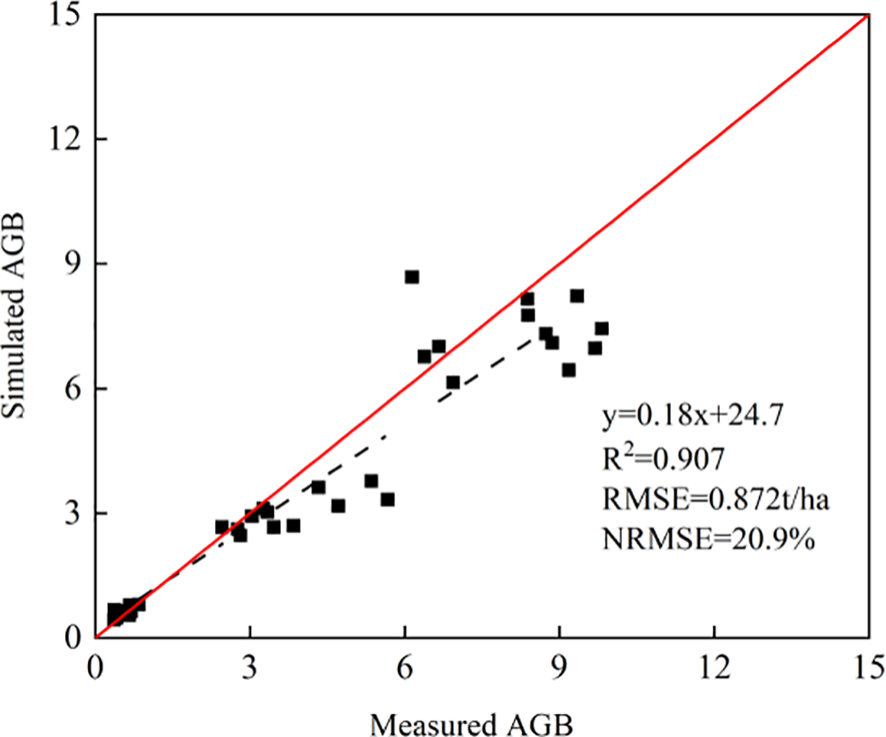
Evaluation of AGB growth accuracy from 2021 to 2023.

As shown in **Figure 6**, the absolute growth monitoring accuracy results using AGB as growth index under different fertilization, seeding and tillage methods from 2021 to 2023 were presented. The R^2^ of the simulated AGB in 2021-2023 is 0.907, and the simulated AGB is in good agreement with the measured AGB. The RMSE is 0.872 t/ha, and the NRMSE is 20.9%.

#### 3.1.3 Comparison of the absolute growth results of LAI and AGB in the key growth period of winter wheat simulated by the model

**Figure 7:**
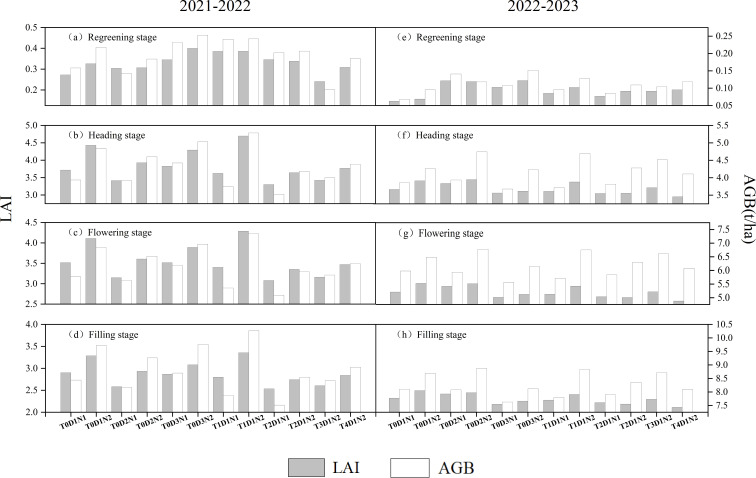
APSIM model simulates the changes in LAI and AGB of winter wheat in four key growth periods of 2021-2022 **(A–D)** and 2022-2023 **(E–H)** under different treatments.

As shown in **Figure 7**, this study compared the variation in LAI and AGB across four key growth stages of winter wheat—regreening, heading, flowering, and filling—over two years and 12 different treatments, using simulations from the APSIM model. The results showed that the changes of LAI and AGB parameters simulated by APSIM model were roughly the same under different treatments in the key growth period of winter wheat. Except for T2D1N2 (normal sowing, plow tillage, and normal fertilization) at the winter wheat regreening stage in 2021-2022 and T0D3N2 (no-tillage, normal sowing, and normal fertilization) at the tasseling, flowering, and irrigating stages of winter wheat in 2022-2023, which did not have the same trend as the neighboring treatments, other treatments showed the results of the same trend of change. The reason for the inconsistent trend of the former may be that winter wheat adjusts its growth to adapt to the environment. Under sufficient fertilization conditions, winter wheat may prioritize root development over leaf growth to adjust to potential nutrient changes. The reason for the inconsistent trend of the latter may be that subsoiling tillage improves soil physical and chemical properties. In contrast, no-tillage may cause soil compaction, hindering root growth and leaf expansion of winter wheat. During key growth stages like heading, flowering, and filling, winter wheat is more sensitive to environmental conditions, making the differences more pronounced. From the perspective of different key growth periods, the regreening stage is characterized by relatively slow growth and recovery due to low temperatures and other limiting factors, resulting in minimal accumulation of LAI and AGB. Consequently, the changes during the regreening period are less noticeable compared to other key growth stages, making it less suitable for distinguishing the effects of different treatments on winter wheat growth.

### 3.2 Relative growth monitoring of winter wheat based on the combination of model simulation parameters and remote sensing inversion parameters

#### 3.2.1 Trend consistency analysis of LAI and NDVI winter wheat relative growth monitoring by remote sensing

**Figure 8:**
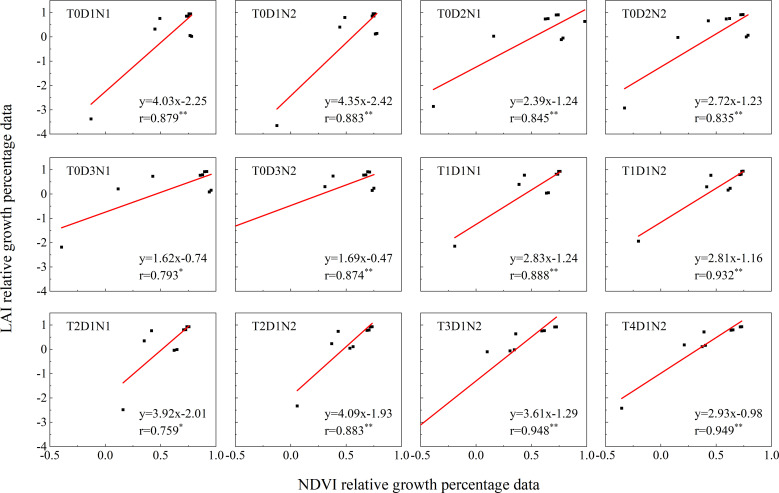
Analysis of NDVI and LAI growth in the study area under different tillage methods, fertilization and sowing treatments from 2021 to 2023. Among them, * indicates significance and ** indicates extremely high significance.

As shown in **Figure 8**, Sentinel-2 data were selected to calculate the NDVI percentage data and LAI percentage data from 2021-2023 in the research area through GEE platform. These two sets of data were then fitted according to different fertilization rates, seeding rates, and tillage methods to monitor and analyze relative growth. The remote sensing NDVI data from 2021 to 2023 and the simulated LAI data were used as growth indicators for data fitting, and the Pearson correlation analysis was performed. The correlation coefficient r value ranged from 0.759 to 0.948. In the case of T4D1N2, the correlation between LAI percentage data and NDVI percentage data was the best, showing a strong correlation of 0.949 (p< 0.001), and T2D1N2 was the worst, showing a strong correlation of 0.759 (p< 0.05). Meanwhile, in terms of significance, except for T2D1N2 and T0D3N1, which showed significance (p< 0.05), all other cases showed highly significant (p< 0.01). Overall, the linear correlation between the two variables was significant, except for T2D1N2 and T0D3N1 which showed a strong correlation, all other cases showed a very strong correlation, all of them had strong linear positive correlation, indicating that it can well reflect the link between LAI and NDVI, and that the fitted correlation between the data in the different cases was better and all of them were significantly positively correlated (p< 0.05).

**Figure 9:**
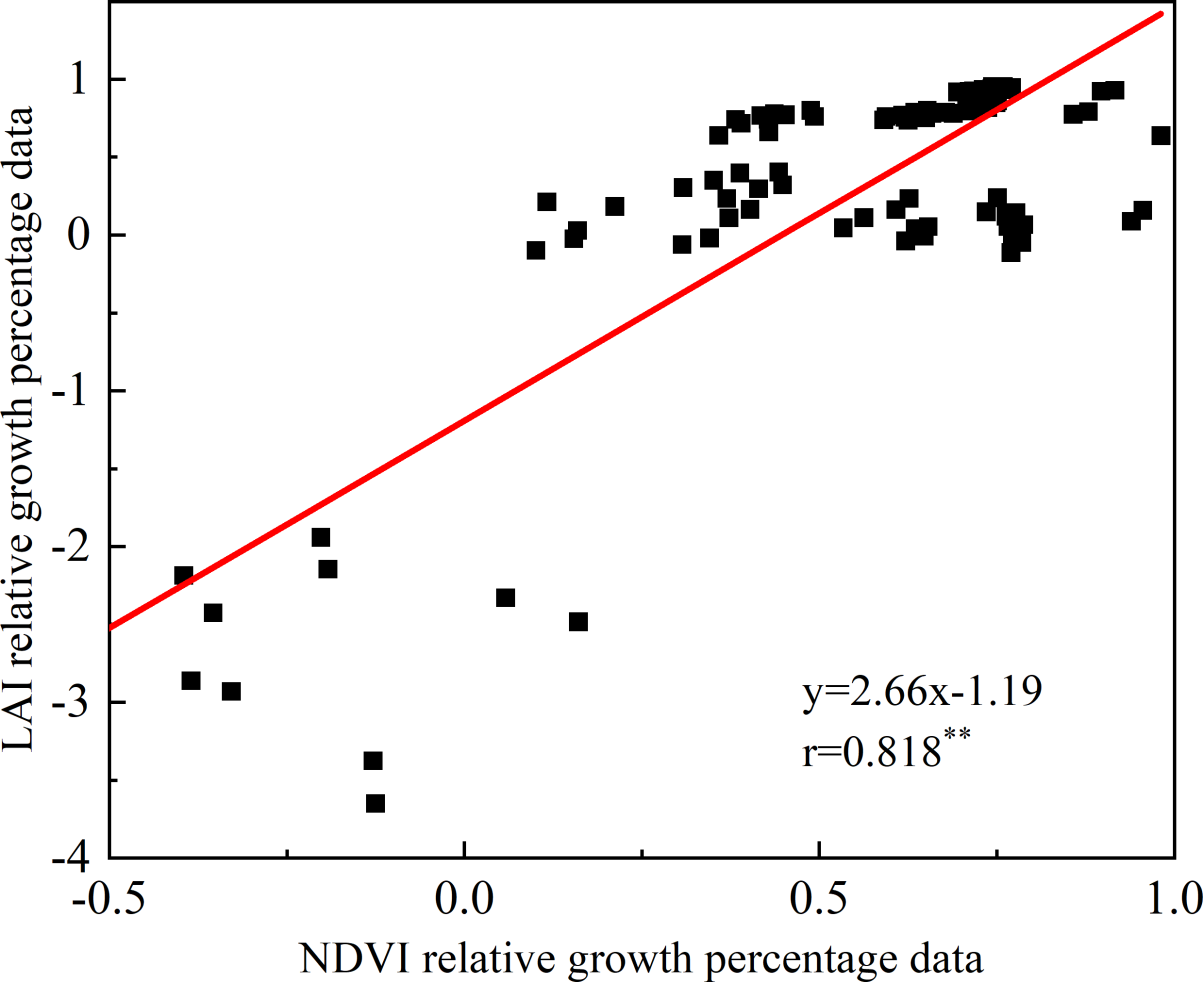
Relative growth analysis of NDVI and LAI from 2021 to 2023. Where “**” indicates the significance test result.

As shown in **Figure 9**, the correlation coefficient r value of the model simulated LAI percentage data and NDVI percentage data in 2021-2023 was 0.818 (p< 0.001), and the simulated LAI percentage data showed a highly significant positive correlation with the NDVI percentage data. In addition, the percentages of NDVI and LAI data, both greater than 0, accounted for about 82%, which suggests that winter wheat growth conditions in 2022 are better than those in 2023.

#### 3.2.2 Trend consistency analysis of AGB and NDVI remote sensing monitoring of relative growth of winter wheat

**Figure 10:**
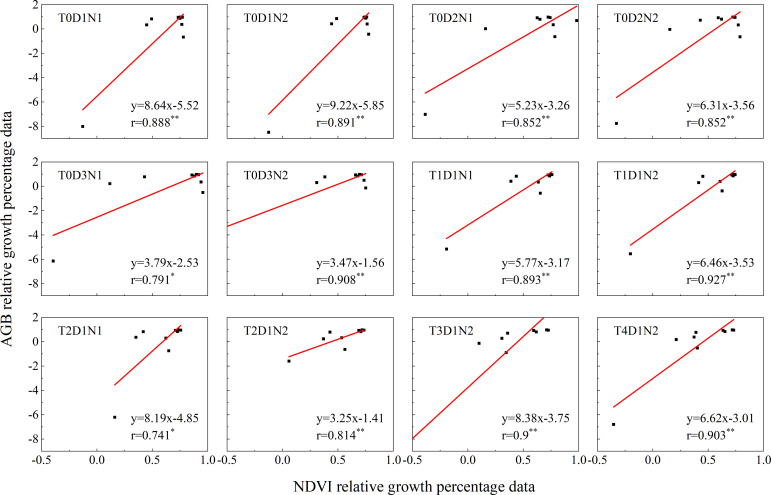
Analysis of NDVI and AGB growth in the study area under different tillage methods, fertilization and sowing treatments from 2021 to 2023. Where “*” and “**” represent significant and extremely significant difference results respectively.

Using the Sentinel-2 data of GEE platform, NDVI percentage data and AGB percentage data from 2021-2023 in the study area were calculated. These two sets of data were then fitted according to different fertilizer application rates, seeding rates, and tillage practices to perform relative growth monitoring. As shown in **Figure 10**. The NDVI data based on the remote sensing and model simulated AGB data from 2021-2023 were fitted to the data as a growth indicator, and Pearson correlation analysis was performed, and the r values ranged from 0.741 to 0.927, and the correlation between the data in different cases was good and all of them showed a positive correlation. The best correlation between the AGB percentage data and the NDVI percentage data was found in the case of T4D1N2, which showed a very strong correlation of 0.927 (p< 0.001), and the worst in the case of T2D1N1, which showed a strong correlation of 0.741 (p< 0.05). Meanwhile, in terms of significance, all cases showed highly significant differences (p< 0.01) except for T2D1N1 and T0D3N1, which showed significant differences (p< 0.05).

**Figure 11:**
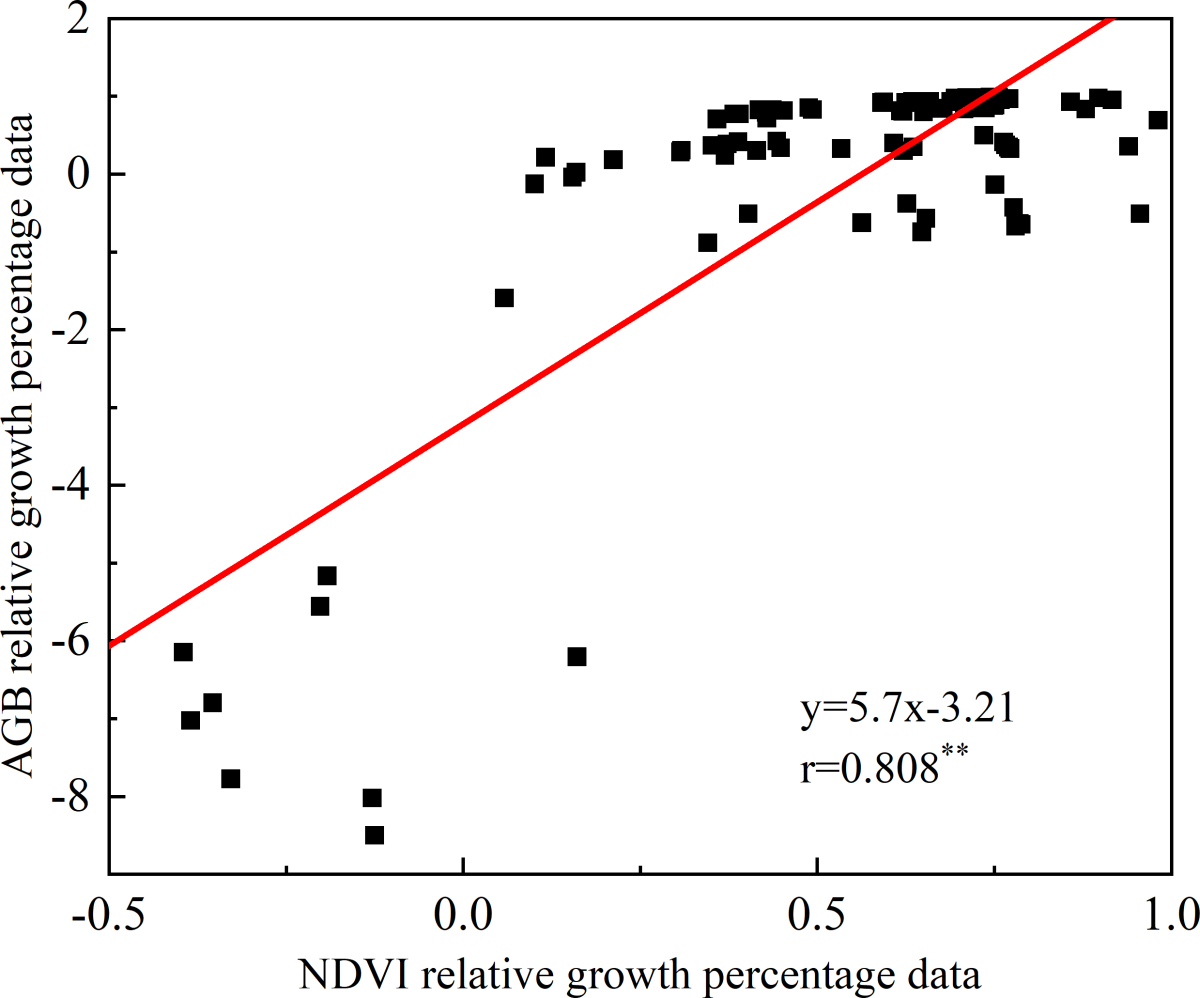
Relative growth analysis of NDVI and AGB from 2021 to 2023. Where “**” indicates the significance test result.

As shown in **Figure 11**, the correlation coefficient r value was 0.808 (P< 0.001) for the simulated AGB difference percentage data and NDVI difference percentage data in 2021-2023, and these data showed highly significant positive correlation. Moreover, the percentage data for which the difference between NDVI and AGB is greater than 0 accounts for about 80%, the AGB results also indicate that the growth conditions for winter wheat in 2022 are better than those in 2023.

## 4 Discussion

### 4.1 The role of APSIM model in monitoring the growth of winter wheat under different treatments and optimizing field management

Crop model can dynamically monitor the growth process of crops (Ren et al., 2011; Xing et al., 2017a). This study shows that the APSIM model shows significant application prospects for monitoring the growth dynamics of winter wheat. In this study, the growth curves of LAI and AGB of winter wheat were obtained. From the LAI growth curve, we can observe that after the wintering period, as the temperature increases, the plants start to grow rapidly, necessitating a significant amount of nutrients and water. Providing appropriate nutrients from the regreening stage to the jointing stage will greatly benefit the growth of winter wheat. Zhao et al. found that the change in NDVI during these two periods was relatively large when extracting winter wheat area by NDVI, so they also put forward this view (Zhao et al., 2011). As LAI started to decline, while AGB continued to increase, it was observed that the growth rate slowed down. This suggests that the leaves of winter wheat are approaching saturation, more energy is being directed towards grain formation, and the photosynthetic efficiency is relatively reduced.

In the analysis of different tillage practices, fertilization, and sowing treatments by using the APSIM model, when two gradients of fertilizer application were applied in this study, we found that other conditions were consistent while different fertilizer application rates were applied, the different treatments showed better growth at the normal fertilizer application rate than at the halved fertilizer application rate, which indicated that the reduction of fertilizer application rate in certain cases had a negative impact on the growth and development of the crop, and that the subsequent study could increase the fertilizer application gradient to determine the optimum fertilizer application rate. Among the treatments with the same other conditions but different sowing rates, 75% of the sowing rate in some treatments of LAI and AGB showed better growth than the other sowing rates, indicating that appropriate reduction in sowing rate can promote wheat growth. When studying the effect of sowing density on the growth and yield of wheat, Xiao et al. found that reducing the sowing rate could stimulate wheat plants to produce more tillers and secondary roots, and promote an increase in plant height, suggesting that appropriate growth density can utilize the available resources, such as sunlight, water, and nutrients, in a more efficient manner (Xiao et al., 2021). Among the treatments with the same other conditions but different tillage methods, the growth of winter wheat was relatively better under deep loosening tillage treatment, and the related research of Feng et al. showed that compared with different tillage methods, subsoiling tillage could significantly increase the organic carbon content in the topsoil and enhance the water holding capacity of the soil, which might be the reason for promoting the growth of winter wheat (Feng et al., 2018). The growth curves of LAI and AGB in different years and under different treatments showed that the growth curves of LAI and AGB in subsoiling tillage, normal sowing, and normal fertilization were better than those in other treatments, and the growth trends of LAI and AGB were significantly greater than those in other treatments, indicating that this treatment may provide more optimized growth conditions, including soil looseness and nutrient supply, etc. these treatments improved the growth environment of plants. Therefore, the most suitable field management methods for winter wheat in these 12 cases are subsoiling tillage, normal sowing, and normal fertilization.

### 4.2 Analysis of correlation results between model simulation parameters and remote sensing inversion parameters

Compared with previous growth monitoring, relative growth remote sensing monitoring relies largely on vegetation indices such as NDVI (Su et al., 2019; Lu et al., 2020) and rarely incorporates other variables. In this study, in addition to NDVI, LAI and AGB are closely related to the growth of winter wheat and are added for trend consistency analysis and are used to comprehensively evaluate the crop’s growth status and changes in the growth status from different perspectives (Zheng et al., 2017; Qin et al., 2022). The comparison between NDVI extracted by Sentinel-2 data with 10m spatial resolution and LAI and AGB data simulated by the model showed that the correlation coefficient r value exceeded 0.74, indicating a positive correlation between LAI and AGB data simulated by NDVI and APSIM models. This is consistent with the obvious positive correlation between NDVI and LAI in different growth periods of winter wheat proposed by Liang et al. (2013) in the inversion of LAI, and the obvious correlation between above-ground biomass and NDVI in the main growth period of winter wheat proposed by Hansen and Schjoerring (2003) in the relevant study. It means that with the increase or decrease of NDVI, LAI and AGB will also increase or decrease to a certain extent.

From the growth consistency results of LAI and AGB and NDVI respectively, the three cases of T1D1N2, T3D1N2, and T4D1N2 showed extremely strong correlation (r > 0.9) in LAI and NDVI and AGB and NDVI, and the worst correlation was also the same both for T2D1N1, which also showed strong correlation. In terms of significance, except for T2D1N2 and T0D3N1, which showed significance (p< 0.05), all other cases showed highly significant (p< 0.01), and the results showed significant correlation, which indicated that correlations due to random factors could be excluded, such as climatic conditions, human activities, and equipment. And it was found that NDVI, AGB and LAI difference percentage data were all greater than 0 accounted for more than 80%, indicating that winter wheat in 2022 under different scenarios grew better than them in 2023. In terms of LAI and AGB structural characteristics, LAI directly reflects the degree of leaf cover and leaf area, leaf density and leaf arrangement of winter wheat plants (Lu et al., 2005), while AGB reflects winter wheat biomass accumulation and plant height, stem thickness, branching status and spike size (Guo et al., 2023). From the results and the structural characteristics of LAI and AGB, the LAI and AGB simulated by the APSIM model can better reflect the growth condition of winter wheat, which is more reliable than relying on NDVI only for relative growth monitoring.

### 4.3 Limitations of APSIM model in winter wheat growth monitoring

From the results of absolute growth of LAI simulated by the APSIM model, the growth simulated during the regreening period was more accurate, but the results of the simulation in the later part of the growth period were on the high side, which may be due to the inaccurate estimation of the model for the process of the decay of the leaf area in the later part of the growth period, or the possible existence of errors and uncertainties in the measured data, for example, due to errors caused by the measurement method, equipment, or sampling errors (Wang and Wang, 2015). In the later research, parameter sensitivity analysis should be combined to determine which parameters have the greatest impact on the accuracy of simulation results. From the absolute growth results of AGB simulated by APSIM model, it can be seen that at the end of the growth period (about 210 d), some of the simulated values are low. Which may be due to the failure of the model to accurately simulate the changes in soil moisture and nutrients and their effects on plant growth, and the simulated biomass may be low, especially in the final stages of crop growth, the supply of water and nutrients has an important impact on plant biomass accumulation (Guo et al., 2016). In later studies, a more precise division of the growth stages in the model is needed, and the model parameters are adjusted according to the characteristics and needs of each stage in order to better capture the sensitivity to water and nutrients at the end of growth. Precision agriculture and smart agriculture are the main development direction of current and future agriculture (Zhao et al., 2021). In the next step, based on the evaluation of the simulation results and the analysis of the shortcomings, we can further improve the APSIM growth model, especially in simulating the leaf area decaying process in the late growth stage and the effects of soil moisture and nutrients on plant growth, or extend the study to other crops or different environmental conditions to verify the generalizability of the study results. This could help agricultural producers and policy makers to understand the growth requirements and optimal management strategies for different crops.

## 5 Conclusions

In this study, the APSIM model and Sentinel-2 data were used to monitor both the relative and absolute growth of winter wheat under various tillage methods, fertilization rates, and sowing treatments. In terms of relative growth monitoring, absolute growth was further validated using remote sensing data, and there was a significant linear positive correlation between NDVI based on the Sentinel-2 data and both LAI and AGB data derived from the APSIM model (r>0.74, p<0.05). It was found that the best correlation with NDVI data (r>0.9, p<0.001) was obtained in the case of normal sowing, normal fertilizer application and rotary tillage. In terms of absolute growth monitoring, the growth of winter wheat was relatively poor in the area where the amount of fertilizer was halved compared to the area with a standard amount of fertilizer. Properly reducing the sowing rate can help to improve the growth of winter wheat. In the case of only different tillage methods, subsoiling tillage shows better growth than other tillage methods. In this paper, compared with other treatments, the most suitable management methods for winter wheat are subsoiling tillage, normal sowing, and normal fertilization. The results showed that the LAI and AGB indicators simulated by the APSIM model could better reflect the growth condition of winter wheat, and were more reliable than relying only on vegetation indices such as NDVI for relative growth monitoring.

## Data Availability

The original contributions presented in the study are included in the article/supplementary material. Further inquiries can be directed to the corresponding authors.
